# The role of the Basic Public Health Service program in the control of hypertension in China: Results from a cross-sectional health service interview survey

**DOI:** 10.1371/journal.pone.0217185

**Published:** 2021-06-18

**Authors:** Jiangmei Qin, Yanchun Zhang, Masha Fridman, Kim Sweeny, Lifang Zhang, Chunmei Lin, Lu Mao

**Affiliations:** 1 China National Health Development Research Center of the National Health and Family Planning Commission, Beijing, China; 2 Victoria Institute of Strategic Economic Studies, Victoria University, Melbourne, Australia; 3 Medical College of Shi Hezi University, Xinjiang, China; Health Services Academy Islamabad Pakistan, PAKISTAN

## Abstract

**Objectives:**

Non-communicable diseases (NCDs) have become the main cause of mortality in China. In 2009, the Chinese government introduced the Basic Public Health Service (BPHS) program to relieve the rising burden of NCDs through public health measures and delivery of essential medical care. The primary aim of this study was to evaluate the impact of the BPHS program on hypertension control.

**Methods:**

The China National Health Development Research Center (CNHDRC) undertook a Cross-sectional Health Service Interview Survey (CHSIS) of 62,097 people from primary healthcare reform pilot areas across 17 provinces from eastern, central, and western parts of China in 2014. The current study is based on responses to the CHSIS survey from 7,867 participants, who had been diagnosed with hypertension. Multi-variable mixed logit regression analysis was used to estimate the association between BPHS management and uncontrolled hypertension. In a follow-up analysis, generalized structural equation modelling (GSEM) was used to test for mediation of the BPHS program effect through patient compliance with medication.

**Findings:**

The estimated proportion of patients with uncontrolled hypertension was 30% lower (23.2% vs 31.5%) in those participants who were adequately managed under the BPHS program. Other predictors of hypertension control included compliance with medication, self-reported wellbeing, income, educational attainment and exercise; smoking was associated with reduced hypertension control. The significant inverse association between uncontrolled hypertension and age indicates poor outcomes for younger patients. Additional testing suggested that nearly 40% of the effect of BPHS management (95% CI: 28.2 to 51.7) could be mediated by improved compliance with medication; there was also an indication that the effect of management was 30% stronger in districts/counties with established digital information management systems (IMS).

**Conclusion:**

Hypertension control improved markedly following active management through the BPHS program. Some of that improvement could be explained by greater compliance with medication among program participants. This study also identified the need to tailor the BPHS program to the needs of younger patients to achieve higher levels of control in this population. Future investigations should explore ways in which existing healthcare management influences the success of the BPHS program.

## Introduction

The leading causes of mortality in China have shifted relatively quickly from infectious diseases and perinatal conditions to chronic diseases and injuries [1,2]. This has been accompanied by an increase in hypertension and other cardiovascular disease risk factors, which were responsible for 2,109,772 deaths from stroke and 1,750,038 deaths from ischemic heart disease in 2017 [3], making these factors the leading causes of death in China. In recognition of the failure of the prevailing health system at the time, the central government introduced a new healthcare reform plan in 2009 to restore the primary healthcare system in both essential medical care and public health service provision [4]. One important measure was the program entitled “Basic Public Health Services” (BPHS) which supports community health organizations to deliver a defined package of basic health services throughout the country [5]. In urban areas, these organizations are called community health centres and stations; in rural areas they are township health centres and village clinics. This essential health care package focuses on maternal and child health, elderly people, and chronic disease patients. A major aim of the program is to combat the increasing burden imposed by non-communicable diseases (NCDs),through a range of measures of management of hypertension and diabetes, including health education, improving medication compliances, control risk factors, such as smoking control, alcohol intake and combating obesity [6], in line with the recommendations by the World Health Organization for essential packages of interventions for non-communicable diseases by primary care facilities [7].

Funding for the program was provided by the Government initially on the basis of 15 Chinese yuan (CNY) per capita each year, which was increased to 20 CNY in 2011 and 50 CNY in 2017 [8]. From 2009 to 2013, over 140 billion CNY (or around USD 21 billion) was invested in this program. It was estimated that about 18% of this investment was spent on management of patients with hypertension for a total of around 25.2 billion CNY (or around USD 3.8 billion) during 2009 to 2013 [9]. By the end of 2013, the number of primary health care facilities providing services under the BPHS reached 2,958,149, including 476,073 community health service centres (or stations), 1,244,054 township hospitals, and 1,238,022 village clinics [10]. The specifications for the BPHS and the requirements for service delivery were revised in 2011, 2013 and 2017, ensuring that hypertensive patients aged 35 and over, be followed-up, monitored, and evaluated on a regular basis. However, little is known about the effectiveness and benefit of such a large investment, especially from the population’s perspective.

A longitudinal cohort study of the Chinese population, the China Health and Retirement Longitudinal Study (CHARLS) conducted in 2015 demonstrated that factors such as age, sex, smoking habits, drinking habits, household income, health insurance, BMI, residential region, marital status, educational level, and nationality were significantly associated with levels of awareness, treatment, and control among people with hypertension [11]. A recent cross-sectional study discovered that a lower likelihood of awareness and treatment of hypertension was associated with younger age, lower income, being male, an absence of previous cardiovascular events, diabetes, obesity, and alcohol use [12]. The rate of control of hypertension was universally low across all subgroups [11]. Data from CHARLS has highlighted the importance of health insurance in the ability of patients to control hypertension [13].

Although primary care in low-resource settings is the backbone for implementation of essential NCD interventions [7,14], very few evaluations have focused on the role of primary care facilities in hypertension control. Two related reports have described the progress of the BPHS program. The Annual Report of Essential Public Health Services Performance Evaluation has been conducted every year since 2010 by the Centre for Project Supervision and Management of the National Health and Family Planning Commission (NHFPC) [15]. It reported that from 2011 to 2015, the number of hypertensive patients managed by the program increased from 65.86 million to nearly 100 million. Another report conducted by the Community Health Association of China (CHAC) in 2014 [16], had similar results to the NHFPC 2013 evaluation. Despite all this, neither of these studies evaluated the effectiveness of the BPHS from the perspective of the population. The BPHS program facilitates patients seeing the same doctor regularly for management of hypertension. Evidence from the USA shows that rates of controlled hypertension were significantly higher among persons who visited the same facility for their health care or saw the same provider [17]. This study aims to identify differences in hypertension control between patients managed by the BPHS program and those not managed by the program by using data from the Cross-sectional Health Service Interview Survey (CHSIS) conducted by the China National Health Development Research Center (CNHDRC). Patients were regarded as having hypertension controlled, if they answered “Yes” to the question “Was your blood pressure normal when it was last measured?”. They were considered as not controlled if the answer was “no” or “not clear”.

12 factors in three categories were considered as determinants of hypertension control in this study. The first category consists of socio-demographic factors influencing adherence to medication, including age, gender, geographical region, health insurance, financial difficulties, and education level. The second category includes disease-related factors, such as smoking, drinking, and exercise. The third factor is hypertension management, denoting medical support and follow-up by a specialist, and is the factor considered as the effect of the BPHS program.

Therefore, this study was to evaluate the effectiveness of the BPHS program in reducing the incidence of uncontrolled hypertension among participants and explore how to optimize the BPHS outcome.

## Methodology

The ethics committee of the China National Health Development Research Centre reviewed and approved the present study, and the written informed consent was obtained from each participant before data collection.

### Objectives

The primary research question in this study was to evaluate the effectiveness of the BPHS program (Management) in reducing the incidence of uncontrolled hypertension among BPHS participants.

The secondary aim of this study was to explore factors which could influence the success of the BPHS program to gain insight into its processes and to provide guidance on ways to optimize BPHS outcomes. This was done by testing the impact of BPHS across demographic and regional groups. We also explored at the mechanism of BPHS action by testing whether the effect of BPHS would be mediated through patient compliance with medication. Finally, we examined the possibility that the success of the BHPS program could also depend on effective integration of existing health services though digital information management systems (IMS), which are currently available in some, but not all districts across China. The results of the secondary analysis are presented in the Supplementary Information and described in the Discussion of this manuscript.

### Source of data and sampling design

The Cross-sectional Health Service Interview Survey (CHSIS) used in this study was conducted in 2014 by the China National Health Development Research Center (CNHDRC) and was funded by the National Health Commission of the People’s Republic of China (NHC) and the China National Natural Science Foundation. The primary objective of the program was to monitor the implementation of primary care reform in China in chosen pilot areas, to use the experience gained to promote the integration of the health care system, and to disseminate this experience across the nation.

Of the 32 provinces in the mainland of China, 17 were chosen to participate in the reform based on their representativeness and willingness to participate, These provinces cover all three regions of Chinal (eastern, central, and western), and the rural-urban divide. One municipality was chosen in each of the 17 provinces as the basis for the program. A municipality in China denotes a large area encompassing both urban areas called urban districts, and rural areas called rural counties. As these are the basic administrative units of the financial and tax system in China, one urban district and one rural county was selected within each of the 17 municipalities, which was also based on their representativeness and willingness to participate. The sampling scheme is described in detail in the Supplementary Information (S1 Table in S1 File).

Most people in urban districts live in an urban setting, although on the outskirts there are people who live on farms, i.e. in rural areas. Similarly, most people in rural counties live on farmland, although the administrative centre of a county is typically urban. Hence, both districts and counties have urban and rural areas. The urban areas are called streets, while the rural areas are called townships.

Multi-stage sampling was used to select 62,097 CHSIS survey participants from 20,777 households, as outlined in Table 1[A] and S1 Table in S1 File (Supplementary Information).

**Table 1:** Sampling schemes for the CHSIS survey [A] and sampling levels for the analysis of Uncontrolled Hypertension [B]. [A] Sampling design for the CHSIS survey. [B] Sampling levels in the analysis of Uncontrolled Hypertension. The sampling scheme for the current study of Uncontrolled Hypertension [B] is derived from the survey design for the BPHS survey [A].

**Sampling levels**		**BPHS program**					
		**Combined**		**Predominantly urban**		**Predominantly rural**	
1. Regions ^a^		3		---		---	
2. Provinces with BPHS programs		17		---		---	
3. Municipalities with BPHS programs		17		---		---	
4. Districts or counties ^b^		34^b^		17 districts		17 counties	
5. Streets or townships							
Total		102		52		50	
per districts or county		3		~3^b^		~3^b^	
6. Neighborhood committees or villages							
Total		408		209		199	
Estimates per street or township		4		~4		~4	
7. Households							
Total		20,777		10,665		10,112	
Mean ± sd [per neighborhood or village		50.9±4.4		51.0±4.1		50.8±4.8	
Range [per neighborhood or village]		20–70		38–70		20–70	
8. Survey respondents							
Total		62,097		30,320		31,777	
Mean ± sd [per household]		3.0±1.3		2.8±1.2		3.1±1.4	
Range [per household]		1–12		1–9		1–12	
Sampling levels		Uncontrolled hypertension among BPHS respondents					
		**Combined**		**Urban**		Rural	
1. Regions		3		---		---	
2. Provinces		17		---		---	
3. Municipalities		17		---		---	
4. Districts or counties		34		17 districts		17 counties	
5. Streets or townships							
Total		102		52		50	
Mean ± sd [per districts or county]		3.0 ± 0		1.97 ± 0.80		1.87 ± 0.51	
Range [per districts or county]		3–3		1–3		1–3	
6. Neighborhood committees or villages							
Total		408		209		199	
Mean ± sd [per street or township]		4.0 ± 0.2		4.0 ± 0.2		4.0 ± 0.1	
Range [per street or township]		3–5		3–5		3–4	
7. Households							
Total		7,867		4,300		3,567	
Mean ± sd [per neighborhood or village]		22.7 ± 7.8		23.9 ± 7.9		21.2 ± 7.5	
Range [per neighborhood or village]		2–42		3–41		2–42	
8. Survey respondents							
Total		9,607		5,338		4,269	
Mean ± sd [per household]		1.22 ± 0.44		1.24 ± 0.45		1.20 ± 0.41	
Range [per household]		1–4		1–4		1–3	
9 Analytical cohort [1 respondent per household]							
Total		7,867		4,300		3,567	
Mean [per household]		1		1		1	
Range		1–1		1–1		1–1	

^a^ Broad regions of China: Eastern, Central and Western China [18].

^b^ In a small number of municipalities, the areas surveyed were exclusively urban (2 districts) or exclusively rural (2 districts).

^c^ The selection of neighborhood committees and villages followed the principle of 4 per street or township strictly. Therefore, in theory there would be 208 neighborhood committee and 200 villages; and therefore, the mean± sd and range are not applicable (N/A) here. However, when implementing the survey one village was replaced by a neighborhood committee within an urban district, because rural villages were much less in number than urban neighborhood committees, which resulted in 209 neighborhood committees and 199 villages.

The survey was carried out using face-to-face interviews in the homes of participants by undergraduates from medical universities trained by CNHDRC personnel. The survey was based on the 5th National Health Interview Survey of Households of China [19], which was conducted in 2013. It was modified in CHSIS to focus on primary health care. The current analysis evaluated responses to questions, which are reproduced in S2 Table in S1 File (Supplementary Information).

Responses from 9,607 CHSIS participants were selected for the current study of hypertension control. Selected participants were aged 15 years and over in the survey answered “Yes” when asked “Have you ever been diagnosed with hypertension by a doctor?” (S2 Table in S1 File). To simplify modelling, a single participant was randomly selected from each household, which had more than one person diagnosed with hypertension. The final number of participants diagnosed with hypertension was 7,867 (Table 1[B]).

### Measurement of hypertension management and control

BPHS criteria for public health service delivery [20] stipulate that primary health care facilities need to screen people aged 35 years or over for hypertension, and once diagnosed, the person should be provided with standard hypertension management. This requires be contacted by a community health care professional at least four times a year, to monitor blood pressure, to check for deterioration in risk factors, and to obtain advice on lifestyle and medication.

In the CHSIS, the question “Have any primary health care workers provided advice for your hypertension control during the past three months?” was specifically designed to evaluate hypertension management under the BPHS program. If the answer was “yes”, the patient’ was considered as being part of the disease management program of the BPHS, and if the answer was “no”, the patient was considered as not being under the disease management program of the BPHS.

Patients were deemed to have controlled hypertension, if they chose “Yes” in response to the question “Was your blood pressure normal when it was last measured?”. Uncontrolled hypertension was assumed if the chosen answer was “no” or “not clear”.

Other questions addressed demographic characteristics (residence, gender, age, number of household participants), socio-economic status (annual disposable income of the household, health insurance, education level), disease-related health behaviors (smoking, drinking and exercise), self-reported wellbeing, and compliance with medication. The questions used to measure these factors are listed in Table 2, with more details provided in the Supplementary Material (S2 Table in S1 File).

**Table 2:** Factors considered in analysis of hypertension control.

Factor	Source	Values
Hukou (Registered residence)	Question^a^ 1	Hukou registration: urban, rural
Gender	Question 2	Male, female
Age	Questions 3,4	Continuous variable from 15 years old.
Education	Question 5	Education level: No education, primary school, junior high school, senior high school, technical school, middle technical school, senior technical school, university or higher
Insurance	Question 6	Health insurance coverage: urban employees’ health scheme, urban residents’ health scheme, new cooperative medical scheme, rural and urban residents’ health insurance, other health insurance
Wellbeing	Question 7	Self-assessed on a scale 0–100.
Smoking	Questions 8,9	None or quit, 1–10, 11–20, 21–30, and 30+ cigarettes per day
Drinking	Question 10	Yes or no
Exercise	Question 11	Once, 1–2, 3–5, and 6 times or over a week
Management	Question 14	Hypertension managed, not managed
Compliance (medication)	Question 15	Compliance with medication: complete compliance, partial compliance, non-compliance
Control	Question 16	Controlled, uncontrolled hypertension
Income	S2 Table in S1 File ^b^	Household annual disposable income: low, lower-middle, middle, upper-middle, upper, extreme upper
Region	S1 Table in S1 File ^b^	Districts and counties grouped into east, central and west regions
District	S1 Table in S1 File	17 urban districts, 17 rural counties
Information Management Systems (IMS)	S5 and S6Tables in S1 File	IMS implementation: districts/counties without IMPS, partial IMS implementation, full IMS implementation,

^a^ BPHS questionnaire.

^b^ Supplementary Information to this manuscript.

### Statistical analysis

#### Primary analysis

The primary aim of the study was to evaluate the impact of appropriate BPHS follow-up (*Management;* Table 2) on hypertension control in the population of CHSIS survey participants, who had a medical history of hypertension. The incidence of *Uncontrolled Hypertension* was used as the outcome measure in this analysis.

Pearson χ^2^ and t-tests were used in a preliminary analysis to flag potential confounders of the association between *Uncontrolled Hypertension* and *Management*. Tests of imbalances in the distribution of known predictors of hypertension across *Management* groups are shown in Table 3.

**Table 3:** Breakdown of demographic characteristics in the study cohort by management groups.

Potential predictors of *Uncontrolled Hypertension*	Not managed [N = 1,584]	Managed [N = 6,283]	Test of difference (P-value)
Hypertension			χ^2^_(1)_ = 51.0 (P<0.001)^a^
Uncontrolled	503 (31.8%)	1450 (23.1%)	
Controlled	1,081 (68.2%)	4,833 (76.9%)	
Compliance			χ^2^_(2)_ = 65.4 (P<0.001)^a^
Every day	1,224 (77.3%)	5,331 (84.9%)	
Sometimes	268 (16.9%)	785 (12.5%)	
Never	92 (5.8%)	167 (2.7%)	
Gender			χ^2^_(1)_ = 5.3 (P = 0.021)^a^
Male	770 (48.6%)	2,851 (45.4%)	
Female	814 (51.4%)	3,432 (54.6%)	
Age			
Mean ± SD	62.5 ± 11.1	63.5 ± 10.8	t = 3.2 (P = 0.002) ^b^
Median [interquartile range]	62 [56–70]	63 [56–71]	χ^2^_(1)_ = 10.9 (P = 0.001) ^c^
Self-reported wellbeing			
Mean ± SD	7.0 ± 1.6	7.1. ± 1.5	t = 1.9 (P = 0.063)^b^
Median [interquartile range]	7 [6–8]	7 [6–8]	χ^2^_(1)_ = 4.1 (P = 0.044)^c^
Education level			χ^2^_(7)_ = 75.7 (P = <0.001)^a^
None	218 (13.8%)	1,240 (19.7%)	
Primary	434 (27.4%)	2,006 (31.9%)	
Junior high	488 (30.8%)	1,725 (27.5%)	
Senior high	209 (13.2%)	687 (10.9%)	
Technical	11 (0.7%)	19 (0.3%)	
Middle technical	91 (5.7%)	226 (3.6%)	
Senior technical	65 (4.1%)	210 (3.3%)	
University or higher	68 (4.3%)	170 (2.7%)	
Income per capita (CNY)			χ^2^_(5)_ = 21.3 (P = 0.001)^a^
0–4,747	91 (5.7%)	485 (7.7%)	
4,748–10,887	387 (24.4%)	1,734 (27.6%)	
10,888–17,631	362 (22.9%)	1,437 (22.9%)	
17,632–26,937	414 (26.1%)	1,388 (22.1%)	
26,938–50,968	287 (18.1%)	1,082 (17.2%)	
>50,968	43 (2.7%)	157 (2.5%)	
Health insurance			χ^2^_(5)_ = 224.3 (P = <0.001)^a^
None	34 (2.2%)	42 (0.7%)	
Urban employee basic	763 (48.2%)	2,275 (36.2%)	
Urban residents	235 (14.8%)	544 (8.7%)	
New cooperative	446 (28.2%)	2,668 (42.5%)	
Urban and rural residents	94 (5.9%)	704 (11.2%)	
Other	12 (0.8%)	50 (0.8%)	
Exercise			χ^2^_(4)_ = 17.9 (P = 0.001)^a^
Never	704 (44.4%)	3,143 (50.0%)	
Up to 1 session/week	65 (4.1%)	205 (3.3%)	
1–2 sessions/week	93 (5.9%)	337 (5.4%)	
3–5 sessions/week	160 (10.1%)	626 (10.0%)	
≥6 sessions/week	562 (35.5%)	1,972 (31.4%)	
Smoking			χ^2^_(4)_ = 4.1 (P = 0.386)^a^
None	1,225 (77.3%)	4,941 (78.6%)	
1–10 per day	120 (7.6%)	507 (8.1%)	
11–20 per day	185 (11.7%)	629 (10.0%)	
21–30 per day	26 (1.6%)	97 (1.5%)	
>30 per day	28 (1.8%)	109 (1.7%)	
Alcohol			χ^2^_(1)_ = 1.1 (P = 0.295)^a^
<3 occasions/week	1,381 (87.2%)	5,538 (88.1%)	
≥3 occasions /week	203 (12.8%)	745 (11.9%)	
Hukou (urban/rural)			χ^2^_(1)_ = 213.6 (P<0.001)^a^
Urban	1,089 (68.7%)	3,030 (48.2%)	
Rural	495 (31.3%)	3,253 (51.8%)	
Residence (urban/rural)			χ^2^_(1)_ = 303.0 (P<0.001)^a^
Urban	1,174 (74.1%)	3,126 (49.8%)	
Rural	410 (25.9%)	3,157 (50.3%)	
Regions (district/county, city)			χ^2^_(33)_ = 875.1 (P<0.001)^a^
1. Xicheng, Beijing	96 (6.1%)	295 (4.7%)	
2. Pinggu, Beijing	76 (4.8%)	215 (3.4%)	
3. Yunhe, Cangzhou	104 (6.6%)	161 (2.6%)	
4. Huanghua, Cangzhou	15 (1.0%)	214 (3.4%)	
5. Yinzhou, Tieling	58 (3.7%)	132 (2.1%)	
6. Xifeng, Tieling	17 (1.1%)	150 (2.4%)	
7. Changning, Shanghai	30 (1.9%)	314 (5.0%)	
8. Pudong, Shanghai	66 (4.2%)	275 (4.4%)	
9. Runzhou, Zhenjiang	52 (3.3%)	207 (3.3%)	
10. Jurong, Zhenjiang	14 (0.9%)	268 (4.3%)	
11. Keqiao, Shaoxing	48 (3.0%)	266 (4.2%)	
12. Shengzhou, Shaoxing	8 (0.5%)	283 (4.5%)	
13. Meilie, Sanming	60 (3.8%)	127 (2.0%)	
14. Shaxian, Sanming	41 (2.6%)	168 (2.7%)	
15. Lanshan, Linyi	43 (2.7%)	108 (1.7%)	
16. Yinan, Linyi	7 (0.4%)	171 (2.7%)	
17. Changyi, Jilin	113 (7.1%)	84 (1.3%)	
18. Panshi, Jilin	15 (1.0%)	159 (2.5%)	
19. Yijiang, Wuhu	96 (6.1%)	126 (2.0%)	
20. Fanchang, Wuhu	38 (2.4%)	182 (2.9%)	
21. Qing Yunpu, Nanchang	108 (6.8%)	133 (2.1%)	
22. Xinjian, Nanchang	15 (1.0%)	88 (1.4%)	
23. Yiling, Yichang	72 (4.6%)	101 (1.6%)	
24. Yidu, Yichang	41 (2.6%)	166 (2.6%)	
25. Yuhu, Xiangtan	31 (2.0%)	237 (3.8%)	
26. Shaoshan, Xiangtan	41 (2.6%)	233 (3.7%)	
27. Hongshan, Chifeng	59 (3.7%)	220 (3.5%)	
28. Keshiketeng, Chifeng	20 (1.3%)	260 (4.1%)	
29. Wuhou, Chengdu	13 (0.8%)	225 (3.6%)	
30. Xinjin, Chengdu	11 (0.7%)	173 (2.8%)	
31. Wudang, Guiyang	62 (3.9%)	96 (1.5%)	
32. Kaiyang, Guiyang	22 (1.4%)	177 (2.8%)	
33. Chengbei, Xining	66 (4.2%)	128 (2.0%)	
34. Huangzhong, Xining	26 (1.6%)	141 (2.2%)	

^a^ Pearson Χ^2^ test. Degrees of freedom are indicated by the subscript.

^b^ T-test.

^**c**^ Kruskal-Wallis equality-of-populations rank test.

Multi-variable mixed logit regression analysis was used to adjust the association between *Management* and *Uncontrolled Hypertension* for possible confounding from known predictors of hypertension. The model also included adjustment for the sampling design (Table 1): the level of *Districts/Counties* was included as an independent variable, while random effects were used to represent clustering at the levels of *Streets/Township* and *Neighborhood /Village*. The contributions of independent variables and random effects to model fit were estimated using Likelihood ratio (LR) tests. The process of model selection is described in detail in S3 Table in S1 File (Supplementary Information).

#### Secondary analysis

The BPHS program is not a single intervention, but a disease management pathway, and any number of practices could be responsible for its effectiveness. The secondary aim of the study was to explore possible mechanisms of BPHS action. One possibility was that improved hypertension control in well-managed patients was due to regular encouragement to take medication. In this scenario the effectiveness of BPHS would be mediated through better compliance with medication (*Compliance*). Generalized Structural Equation Modelling (GSEM) was used to estimate direct and indirect (mediated) effects of Management, as described in S1 Fig and S4 Table in S1 File (Supplementary Information).

Another possibility is that the effectiveness of the BPHS program could be the result of improved access to more intensive medical treatment for patients with failed hypertension control. With regular follow-up, patients with uncontrolled hypertension could be flagged and referred to specialist medical facilities. Such large-scale patient follow-up would rely on efficient information management. Digital information management systems (IMS) have been only partially implemented in China, as shown in S5 Table in S1 File (Supplementary Information). The importance of IMS to the success of the BPHS program was explored by comparing *Management* rate ratios between districts/counties fully established IMS, partially established IMS, or no IMS (S5 and S6 Tables in S1 File, Supporting Information). *Management* rate ratios for each IMS group were derived from three separate multi-variable mixed logit regression models of Uncontrolled Hypertension (S6 Table in S1 File).

All analysis in this report was carried out using Stata 14.1.

## Results

### Demographic characteristics

Key demographic characteristics for managed and unmanaged survey participants are described in Table 3. These characteristics were quite similar for both management groups (not managed vs managed): *Compliance* with medication every day (77.3% vs 84.9%), male *Gender* (48.6% vs 45.4%), *Age* (62.5 vs 63.5 years), *Self-reported wellbeing* (7.0% vs 7.1%), *Smoking* (22.7% vs 21.4%) and frequent *Alcohol* intake (12.8% vs 11.9%). There were also broad similarities in proportions across categories of *Education*, *Income* and *Exercise*. Even though strong statistical differences between management groups were detected for most of these characteristics, such differences arose not from meaningful disparities, but from the large sample size (N = 7,867), which made tests of significance sensitive to relatively minor differences.

Marked differences in *Management* were observed among geographical characteristics: urban *Hukou* (68.7% vs. 48.2%), urban *Residence* (74.1% vs. 49.8%) and in the breakdown by *District/County* (Table 3). There were also differences in categories of *Health Insurance*, which are closely related to place of residence.

### Variable selection for the model of *Uncontrolled Hypertension*

Levels of *Uncontrolled Hypertension* in managed and unmanaged groups were compared using multivariate regression modelling to adjust for potentially confounding population imbalances in the key characteristics that were listed in Table 1. Variable selection for inclusion in the regression model is summarized in Table 4 and the process is outlined in S3 Table in S1 File (Supplementary Information).

**Table 4:** Selection of variables for inclusion in multi-variable mixed logit regression models designed to test the association between *Management* and *Uncontrolled Hypertension*.

Potential predictors of *Uncontrolled Hypertension*	Model contributions LR test χ2 (p-value)	d.f. (Degree of freedom)
**Key variable of interest**		
• Management	44.7 (p<0.001)	1
**Potential confounders**		
• District	227.0 (p<0.001)	33
• Income	26.1 (p<0.001)	5
• Compliance	81.2 (p<0.001)	2
• Exercise	12.9 (p = 0.012)	4
• Education	29.5 (p<0.001)	7
• Wellbeing	40.1 (p<0.001)	1
• Age	8.4 (p = 0.004)	1
• Smoking	10.6 (p = 0.031)	4
**Random effects**		
• Street / Township clusters (ICC ^a^ ± s.e.)	9.0e^-35^ ± 7.4e^-19^	1
• Neighbourhood committee / Village (ICC ^a^ ± s.e.)	0.19 ± .04	1
• LR test [χ2 (p-value)]	46.6 (p<0.001)	1
**Full model**		
• Combined predictors	1,235.0 (p<0.001)	59
• Sample size	7,867	---
**Variables excluded from the final model**		
• Alcohol	2.9 (p = 0.089)	1
• Gender	1.0 (p = 0.327)	1
• Insurance	3.7 (p = 0.593)	5
• Hukou	1.4 (p = 0.245)	1
• Urban / Rural residence	<0.1 (p = 0.899)	1

^a^ Intra-class correlation.

*Management* was one of three most influential independent predictors of hypertension control (LR test: χ^2^_(1)_ = 44.7), along with *Compliance* (χ^2^_(1)_ = 81.2) and *Region* (χ^2^_(33)_ = 227.0). The significance of random effects (χ^2^_(1)_ = 46.6, Table 4) indicated a tendency for clustering among cases of *Uncontrolled Hypertension* at the level of *Neighbourhood committee /Village*.

Table 4 also lists independent variables, which were tested, but which did not significantly improve the overall fit of the regression model: *Alcohol*, *Gender*, *Insurance*, *Hukou*, or *Urban/rural Residence*. These variables were excluded from the final model.

### Predicted impact of BPHS management on hypertension control

The results of regression modelling (Table 5) predicted a 26% reduction in *Uncontrolled Hypertension* among adequately managed participants (23.2% vs 31.5%). The benefit of Management was highly significant (P<0.001), despite adjustment for Compliance, which was included in the regression model as an independent variable. This finding suggested that Management offered a benefit that was additional to the benefit of improved Compliance with medication.

**Table 5:** Estimated effect of management from regression model.

Independent variables	Odds ratio [95% CI] ^a^	Predicted proportions [95% CI]	Predicted change in Uncontrolled Hypertension	
			Risk ratio [95% CI]	P-value
**Management**				
• Not managed	Reference	31.5% [28.8–34.2]	Reference	Reference
• Managed	0.60 [0.52–0.70]	23.2% [21.8–24.6]	0.74 [0.68–0.80]	<0.001
**Compliance**				
• Every day	Reference	22.9% [21.7–24.1]	Reference	Reference
• Sometimes	1.66 [1.41–1.94]	31.2% [28.6–33.9]	1.36 [1.24–1.50]	<0.001
• Never	3.00 [2.24–4.00]	42.4% [36.8–48.1]	1.85 [1.61–2.13]	<0.001
**Wellbeing (**change/10%) ^b^	0.88 [0.85–0.92]	trend shown in Fig 1	0.93 [0.91–0.95] ^b^	<0.001
**Age (**change/10 years of age) ^c^	0.91 [0.86–0.97]	trend shown in Fig 1	0.95 [0.91–0.98] ^c^	0.003
**Educational attainment**				
• None	Reference	27.9% [25.5–30.3]	Reference	Reference
• Primary	0.94 [0.80–1.11]	26.9% [25.1–28.7]	0.96 [0.87–1.06]	0.479
• Junior high	0.76 [0.63–0.92]	23.6% [21.8–25.5]	0.85 [0.75–0.95]	0.005
• Senior high	0.62 [0.48–0.79]	20.5% [17.8–23.3]	0.74 [0.63–0.87]	<0.001
• Technical	0.31 [0.09–1.10]	12.5% [0.4–24.6]	0.45 [0.17–1.18]	0.106
• Middle technical	0.56 [0.39–0.80]	19.2% [14.8–23.6]	0.69 [0.54–0.88]	0.003
• Senior technical	0.75 [0.51–1.12]	23.5% [18.1–28.8]	0.84 [0.66–1.08]	0.172
• University or higher	0.52 [0.32–0.84]	18.3% [12.5–24.1]	0.66 [0.47–0.92]	0.013
**Income per capita (Yuan)**				
• 0–4,747	Reference	30.7% [27.1–34.2]	Reference	Reference
• 4,748–10,887	0.81 [0.66–1.01]	27.2% [25.3–29.1]	0.89 [0.80–0.98]	0.017
• 10,888–17,631	0.67 [0.53–0.84]	24.1% [22.1–26.0]	0.83 [0.74–0.93]	0.001
• 17,632–26,937	0.60 [0.47–0.77]	22.5% [20.4–24.5]	0.82 [0.71–0.95]	0.006
• 26,938–50,968	0.60 [0.45–0.78]	22.3% [19.6–25.0]	0.66 [0.45–0.95]	0.026
• >50,968	0.43 [0.25–0.74]	17.8% [11.3–24.3]	0.58 [0.40–0.85]	0.005
**Exercising**				
• Never	Reference	26.6% [25.1–28.1]	Reference	Reference
• up to 1 session/week	0.91 [0.64–1.27]	25.0% [19.8–30.2]	0.94 [0.76–1.16]	0.571
• 1–2 sessions/week	0.82 [0.62–1.08]	23.5% [19.4–27.5]	0.88 [0.74–1.06]	0.171
• 3–5 sessions/week	0.79 [0.63–0.99]	22.9% [19.9–26.0]	0.86[0.75 - <1.00]	0.031
• ≥6 sessions/week	0.78 [0.66–0.90]	22.7% [20.8–24.5]	0.85 [0.78–0.94]	0.001
**Smoking (number of cigarettes)**				
• none	Reference	24.5% [23.3–25.8]	Reference	Reference
• 1–10 per day	0.95 [0.76–1.18]	23.7% [20.5–26.9]	0.97 [0.84 to 1.11]	0.617
• 11–20 per day	1.10 [0.91–1.32]	26.0% [23.2–28.8]	1.06 [0.95 to 1.19]	0.316
• 21–30 per day	1.69 [1.11–2.57]	33.3% [25.8–40.8]	1.36 [1.08 to 1.70]	0.009
• >30 per day	1.52 [1.02–2.27]	31.4% [24.5–38.3]	1.28 [1.02 to 1.60]	0.030
**Regions**				
1. Xicheng, Beijing	Reference	10.1% [05.7–14.5]	Reference	Reference
2. Pinggu, Beijing	4.42 [2.37–8.24]	30.7% [23.7–37.8]	3.04 [1.87–4.97]	<0.001
3. Yunhe, Cangzhou	1.00 [0.49–2.04]	10.1% [05.9–14.4]	1.00 [0.55–1.83]	0.994
4. Huanghua, Cangzhou	2.00 [1.02–3.95]	17.7% [11.8–23.6]	1.75 [1.01–3.05]	0.046
5. Yinzhou, Tieling	5.54 [2.91–10.55]	35.3% [27.0–43.5]	3.49 [2.14–5.71]	<0.001
6. Xifeng, Tieling	6.07 [3.12–11.80]	37.2% [28.4–45.9]	3.68 [2.23–6.07]	<0.001
7. Changning, Shanghai	1.20 [0.59–2.43]	11.8% [06.7–16.8]	1.16 [0.64–2.11]	0.616
8. Pudong, Shanghai	1.33 [0.68–2.60]	12.8% [08.2–17.4]	1.27 [0.72–2.22]	0.406
9. Runzhou, Zhenjiang	1.15 [0.56–2.36]	11.3% [06.4–16.3]	1.12 [0.61–2.07]	0.708
10. Jurong, Zhenjiang	2.06 [1.07–3.98]	18.1% [12.4–23.8]	1.79 [1.05–3.07]	0.033
11. Keqiao, Shaoxing	2.45 [1.29–4.63]	20.6% [14.8–26.3]	2.04 [1.21–3.42]	0.007
12. Shengzhou, Shaoxing	1.43 [0.71–2.85]	13.6% [08.4–18.7]	1.34 [0.76–2.39]	0.315
13. Meilie, Sanming	3.73 [1.93–7.21]	27.6% [20.0–35.2]	2.73 [1.63–4.57]	<0.001
14. Shaxian, Sanming	3.10 [1.60–5.99]	24.3% [17.4–31.3]	2.41 [1.43–4.07]	0.001
15. Lanshan, Linyi	4.76 [2.42–9.40]	32.3% [23.5–41.0]	3.19 [1.91–5.34]	<0.001
16. Yinan, Linyi	7.99 [4.15–15.40]	43.1% [34.2–51.9]	4.27 [2.63–6.94]	<0.001
17. Changyi, Jilin	5.42 [2.85–10.30]	34.8% [26.7–42.9]	3.45 [2.10–5.64]	<0.001
18. Panshi, Jilin	3.37 [1.72–6.59]	25.7% [18.2–33.3]	2.55 [1.51–4.32]	<0.001
19. Yijiang, Wuhu	1.54 [0.78–3.05]	14.4% [09.2–19.7]	1.43 [0.81–2.52]	0.213
20. Fanchang, Wuhu	2.05 [1.05–4.01]	18.0% [12.2–23.9]	1.78 [1.03–3.08]	0.038
21. Qing Yunpu, Nanchang	4.97 [2.63–9.37]	33.0% [25.3–40.8]	3.27 [2.00–5.35]	<0.001
22. Xinjian, Nanchang	5.38 [2.63–11.04]	34.7% [24.6–44.8]	3.43 [2.02–5.84]	<0.001
23. Yiling, Yichang	4.13 [2.13–7.99]	29.4% [21.6–37.3]	2.92 [1.75–4.87]	<0.001
24. Yidu, Yichang	2.27 [1.16–4.44]	19.5% [13.3–25.7]	1.93 [1.12–3.31]	0.017
25. Yuhu, Xiangtan	3.40 [1.79–6.46]	25.9% [19.0–32.8]	2.57 [1.55–4.27]	<0.001
26. Shaoshan, Xiangtan	4.20 [2.23–7.91]	29.8% [22.7–36.9]	2.95 [1.80–4.84]	<0.001
27. Hongshan, Chifeng	3.17 [1.68–5.99]	24.7% [18.4–31.1]	2.45 [1.47–4.07]	0.001
28. Keshiketeng, Chifeng	13.05 [6.97–24.44]	54.0% [45.9–62.1]	5.35 [3.37–8.49]	<0.001
29. Wuhou, Chengdu	1.58 [0.76–3.26]	14.7% [08.5–20.9]	1.46 [0.80–2.65]	0.216
30. Xinjin, Chengdu	1.23 [0.58–2.60]	12.0% [06.6–17.4]	1.19 [0.64–2.23]	0.582
31. Wudang, Guiyang	4.16 [2.12–8.18]	29.6% [21.2–38.1]	2.93 [1.74–4.93]	<0.001
32. Kaiyang, Guiyang	5.74 [2.98–11.03]	36.0% [27.6–44.3]	3.56 [2.17–5.86]	<0.001
33. Chengbei, Xining	4.79 [2.51–9.13]	32.3% [24.3–40.3]	3.20 [1.95–5.26]	<0.001
34. Huangzhong, Xining	7.29[3.77–14.13]	41.1% [32.1–50.0]	4.07 [2.49–6.65]	<0.001

^a^ 95% confidence intervals.

^b^ Estimate of change at 60% self-reported wellbeing vs. 50% self-reported wellbeing (see Fig 1).

^c^ Estimate of change at 60 years of age vs. 50 years of age (see Fig 1).

**Fig 1:**
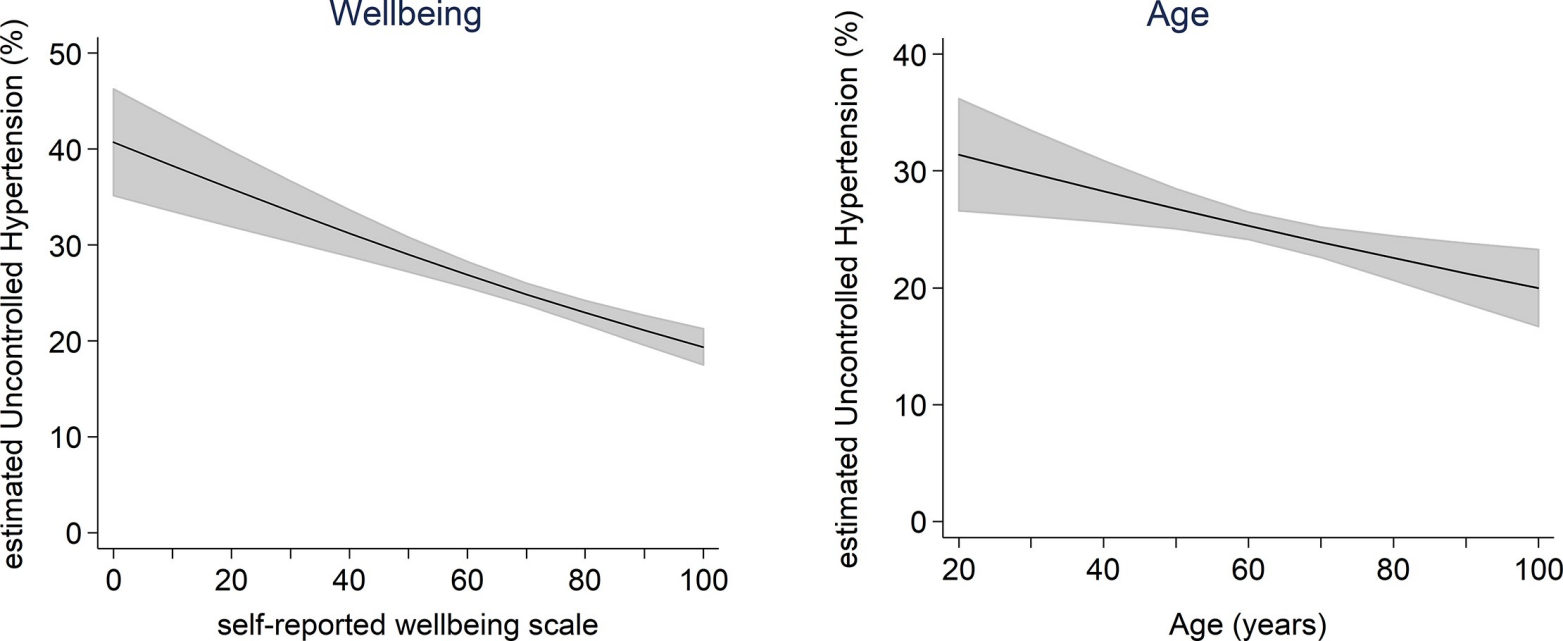
Predicted association between Uncontrolled Hypertension and two continuous variables, Wellbeing (left) and Age (right). The trends were estimated using a mixed logit regression model of Uncontrolled Hypertension. Summary measures of these associations are listed in Table 5: the level of Uncontrolled Hypertension was predicted to decline by 7% with a 10% increase in self-reported Wellbeing (p<0.001), and by 5% with a 10-year increase in age (p = 0.003). Shaded areas represent 95% confidence intervals.

The significance of the inverse association between uncontrolled hypertension and age points to poor hypertension control in younger people (Fig 1). The trend is consistent with previous studies in both China and the USA, which showed that younger adults were less likely to be treated for hypertension than older adults [21,22].

The effects of most other independent variables were in line with expectations (Table 5). Predictors of improved hypertension control included *Compliance*, self-reported *Wellbeing*, *Income*, *Educational attainment* and three or more *Exercise* sessions per week, while smoking over 20 cigarettes per day was associated with reduced control. Levels of control also varied between *Regions* (Table 3), with the most favourable outcomes predicted for Xicheng (10.1% *Uncontrolled Hypertension*), Yunhe (10.1%), Runzhou (11.3%), Changning (11.8%), Wuhou (14.7%), Yijiang (14.4%), Pudong (12.8%), Shengzhou (13.6%) and Xinjin (12.0%); while the least favourable were in Keshiketeng (54.0%) and Yinan (43.1%).

## Discussion

This study examined the relationship between hypertension control and a disease management program, which was provided through the BPHS using data from the CHSIS. The current report provides strong evidence supporting the effectiveness of the BPHS program, which was associated with a 26% reduction in the incidence of Uncontrolled Hypertension.

A number of factors could have contributed to the success of the program, since it does not consist of a single intervention, but a process of patient monitoring and follow-up. This study explored possible reasons behind the effectiveness of the BPHS program, in order to gain insight into its processes and to provide guidance for future efforts to optimize its outcomes.

One possible explanation is that regular face-to-face follow-up with health professionals may encourage improved compliance with medication. The results of mediation analysis (S1 Fig and S4 Table in S1 File, Supplementary Information) showed that that nearly 40% (95% CI: 28.2 to 51.7) of the effect of Management could be explained by improved Compliance with medication among patients, who were managed through the BPHS program. The BPHS program encourages patients to visit the same care provider regularly. Internationally, consistently visiting the same facility for health care or seeing the same provider is conducive to medication compliance and hence improved hypertension control [23].

Another possible driver behind the effectiveness of the BPHS program in controlling hypertension is the integration of the program with other medical services, including timely referral of patients with uncontrolled hypertension to higher level medical facilities. Appropriate referral for more intensive testing and treatment in a hospital or a specialist clinic is particularly important in the treatment of more severe hypertension or secondary hypertension, caused by diabetes, kidney disease or heart disease [24]. Effective information management is required in this scenario to to flag patients with failed hypertension control, then follow up with a referral for treatment [20]. The process of referral and follow-up could be expected to be more efficient in districts/counties with established digital information management systems (IMS). Not all regions in China are covered by IMS; a breakdown of IMS coverage for urban and rural regions in each district/county is reproduced in S4 Table in S1 File (Supplementary Material). The effect of Management was nearly 30% stronger in districts/counties with fully established IMS compared with districts without IMS (Management RR: 0.69 vs. 0.97, P-value = 0.017; S6 Table in S1 File, Supplementary Material). This finding suggests that the effectiveness of the BPHS program may depend on effective information management.

Many of the studies on the control of hypertension give prominent roles to behavioral factors, including smoking, alcohol drinking and exercise, as well as socio-economic factors, such as education, income and insurance, while taking healthcare accessibility and availability into account [6,25]. The guidelines for BPHS service delivery require personnel to “Conduct health education for all patients to encourage life style behavioral improvement objectives and evaluate the progress in follow-up visits, letting patients know when to visit doctors immediately” [26,27]. However, in this study, these factors were not as influential in the control of hypertension as the management provided by the BPHS.

The inverse association between uncontrolled hypertension and age was observed in a previous study by Li *et al*. in 2017 [28]. The trend may be due to a more general failure among younger people to seek medical care, including treatment for conditions, which may cause hypertension such as diabetes and chronic kidney disease [29,30]. There could be several explanations for this reluctance to seek treatment. Many young people fail to appreciate the risks of uncontrolled hypertension [31]. They are also more likely to be part of a “floating population”, which derives little benefit from the health insurance system [32,33]. In China, health insurance is administered at the level of local government, and the floating population does not have access to it.

Policy implications from the study are threefold. Firstly, this study estimated impact of the BPHS program in improving hypertension control at the national level. Secondly, the inverse relationship between uncontrolled hypertension and age suggests that future BPHS policy initiatives should to address the need for younger patients to achieve higher levels of control [3,29]. Thirdly, the BPHS program could provide even more benefit if existing healthcare management processes, such as information management, could be optimized.

The wide scope of this study is one of its main strengths. It is based on a nation-wide survey, designed to evaluate the performance of the BPHS program throughout China. The survey instrument was based on a well-established questionnaires design, widely used for national health surveys in China and worldwide. The survey also collected information on all relevant determinants of hypertension control, which had been identified in previously published studies. Our sample size was large enough to be fully representative of the hypertensive population in China and to enable robust statistical analysis.

Limitations include reliance on cross-sectional survey data, which was collected at a single time point, and which may not be able to establish causality, in particular the relationship between the management provided by the BPHS program and hypertension control. However, cross-sectional surveys do provide important clues for causal studies. A further limitation is that the measure of hypertension control of hypertension relied on self-reporting, with no verification from clinical records or additional blood pressure measurement, While self-reporting may be subject to recall bias, it should be minimised in this study, as over 88% of respondents had blood pressure tested within a month before the survey.

## Conclusions

The BPHS program delivered through local government organizations have played a significant role in improving the control of hypertension and providing a greater degree of equalization of healthcare service delivery, both between urban and rural residents and among different socio-economic groups in China since its introduction in 2009. Some of that improvement could be explained by greater compliance with medication among program participants. The effect of Management appeared stronger in districts/counties with fully established IMS, suggesting the outcomes from the BPHS program could be further enhanced through effective health information management. Lastly, implementation of the BPHS program needs to address the requirements of younger patients to improve the management of hypertension control in this population.

## Supporting information

S1 File (DOCX)

S1 Data (CSV)
