# Correction: The role of the Basic Public Health Service program in the control of hypertension in China: Results from a cross-sectional health service interview survey

**DOI:** 10.1371/journal.pone.0305660

**Published:** 2024-06-14

**Authors:** Jiangmei Qin, Yanchun Zhang, Masha Fridman, Kim Sweeny, Lifang Zhang, Chunmei Lin, Lu Mao

There is an error in the affiliation for authors Jiangmei Qin, Yanchun Zhang, Lifang Zhang, and Chunmei Lin. The correct affiliation is: China National Health Development Research Center, Beijing, China.
