# Supplementary material for: The role of the Basic Public Health Service program in the control of hypertension in China: Results from a cross-sectional health service interview survey
Source: PLoS One. 2021 Jun 18;16(6):e0217185. doi: 10.1371/journal.pone.0217185 (PMC8213048; doi:10.1371/journal.pone.0217185)
Supplement: S1 File — (DOCX) [file pone.0217185.s001.docx]

**Supporting information**

**S1 Table. Regions, municipalities, districts, counties, streets and townships sampled for survey.**

| **Regions** | **Province** | **Municipality** | **District or county** | **Streets** | **Townships** | **Number of streets and townships** |
| --- | --- | --- | --- | --- | --- | --- |
| East | Beijing | Beijing | 1.Xicheng District | Yuetan, Desheng, and Taoranting |  | 3 streets |
|  |  |  | 2.Pinggu District (rural) | Binhe | Jinhaihu and Huangsongyu | 1 street+2 townships |
|  | Hebei | Cangzhou | 3.Yunhe District | Nanhuan, Nanhu | Nanchentun | 2 streets+1 township |
|  |  |  | 4.Huanghua County | Huazhong | Guanzhuang, Luqiao | 1 street+2 townships |
|  | Liaoning | Tieling | 5. Yinzhou District | Hongqi , Tongzhong , Tiexi |  | 3 streets |
|  |  |  | 6. Xifeng County | Xifeng Town | Gaojiadian, Anmin | 1 street+2 townships |
|  | Shanghai | Shanghai | 7. Changning District | Jiangsu Road, Zhoujiaqiao | Xinjing | 2 streets+1 township |
|  |  |  | 8. Pudong District, Shanghai | Dongming Road | Hangtou, Huinan | 1 street+2 townships |
|  | Jiangsu | Zhenjiang | 9. Runzhou District | Baota Road, Jinshan, Jiangqiao |  | 3 streets |
|  |  |  | 10. Jurong County | Chongming | Guozhuang, Biancheng | 1 street+2 townships |
|  | Zhejiang | Shaoxing | 11. Keqiao District | Huashe | Anchang, Xialv | 1 street+2 townships |
|  |  |  | 12.Shengzhou County | Shanhu | Ganlin, Jinting | 1 street+2 townships |
|  | Fujian | Sanming | 13. Meilie District | Liexi, Xubi | Yangxi | 2 streets+1 township |
|  |  |  | 14. Sha County | Fenggang | Xiamao, Nanyang | 1 street+2 townships |
|  | Shandong | Linyi | 15. Lanshan District | Jinqueshan, Yinqueshan | Fangcheng | 2 streets+1 township |
|  |  |  | 16. Yinan County | Jiehu | Andi, Xinji | 1 street+2 townships |
| Central | Jilin | Jilin | 17. Changyi District | Xinghua, Dongjuzi | Huapichang | 2 streets+1 township |
|  |  |  | 18. Panshi County | Henan | Yantongshan, Baoshan | 1 street+2 townships |
|  | Anhui | Wuhu | 19. Yijiang District | Yijiangqiao, Baima, Nanrui Dang Gongwei |  | 3 streets |
|  |  |  | 20. Fanchang County | Fanyang | Pingpu, Hengshan public Service Centre | 1 street+2 townships |
|  | Jiangxi | Nanchang | 21.Qingyunpu District | Jingshan, Hongdu | Qingyunpu | 2 streets+1 township |
|  |  |  | 22. Xinjian County |  | Xishan, Lianhe, Lehua | 3 townships |
|  | Hubei | Yichang | 23. Yiling District | Xiaoxita | Taipingxi, Fenxiang | 1 street+2 townships |
|  |  |  | 24. Yidu County | Lucheng | Gaobazhou, Wangjiafan | 1 street+2 townships |
|  | Hunan | Xiangtan | 25. Yuhu District | Zhongshan, Guangchang | Changcheng | 2 streets+1 township |
|  |  |  | 26. Shaoshan County | Qingxi | Shaoshan, Yintian | 1 street+2 townships |
| West | Inner Mongolia | Chifeng | 27. Hongshan District | Xicheng , Qiaobei | Hongmiaozi | 2 streets+1 township |
|  |  |  | Keshiketengqi County | Xilamulun | Yuzhoudi Dalainuori | 1 street+2 townships |
|  | Sichuan | Chengdu | 29. Wuhou District | Yulin, Hongpailou, Jitou Qiao |  | 2 streets+1 township |
|  |  |  | 30. Xinjin County | Wujin | Xingyi, puxing | 1 street+2 townships |
|  | Guizhou | Guiyang | 31. Wudang District | Xintian, Gaoxin | Xinchang | 2 streets+1 township |
|  |  |  | 32. Kaiyang County | Chengguan | Shuangliu , Nanlong | 2 streets+1 township |
|  | Qinghai | Xining | 33. Chengbei District | Xiaoqiaodajie, Mafang | Dabaozi | 2 streets+1 township |
|  |  |  | 34.Langzhong County |  | Xibao, Lanlongkou, Gonghe | 3 townships |
| Total | 17 provinces | 17 municipalities | 34 districts and counties | 52 streets | 50 townships |  |

**Detailed survey framework with examples**

Table S1 above lists the municipalities, districts and counties, streets and townships included in the survey. The multi-stage, stratified and cluster random sampling framework for the survey has been described earlier and it is summarised in Table 1 of the manuscript.

. Here we set out how the sampling was undertaken for Hebei province.

In the mainland China, there is a hierarchical structure of administrative management. There are 32 provincial regions in mainland China, one among which is Hebei province, one of the 17 provinces in the survey. There are 11 municipalities in Hebei province, and one of them called Cangzhou was selected.

Municipalities are further divided into urban districts and rural counties. In the municipality of Cangzhou, there are 2 urban districts and 14 rural counties. From the 2 urban districts, we selected Yunhe district for this survey and from the 14 rural counties, we selected Huanghua county.

Urban districts and rural counties are further divided into streets and townships respectively. In the district of Yunhe, there are 6 streets and 2 townships. We selected two streets called Nanhuan and Nanhu, and one township called Nanchentun.

Streets are divided into neighborhood committees and townships into villages. In this study, we selected 4 neighborhood committees in each street and 4 villages in each township.

Here we provide more detail of the selection of streets, townships, neighborhood committees and villages, households and respondents within households.

1. **The selection of streets and townships**

Under guidance from the research team, the administrative health department from each district or county carried out the selection of streets and townships.

They were instructed to select streets and townships according to the proportion of local urban and rural population. In principle, two sample streets and one sample township were selected from districts dominated by urban population; one sample street and two sample townships were to be allocated from counties dominated by rural population; and three sample streets were to be allocated for districts without a rural population. The townships and streets were to be selected randomly (e.g. draw lots)

1. **The selection of neighbourhood committees and villages**

Four neighborhood committees were randomly selected within each street. Four villages within each township were selected with the following constraint: one of the villages had to be the seat of the township government. Next, 50 households were randomly selected from each neighborhood committee and each village.

The neighborhood committees were randomly selected by district-level health departments and the selection of buildings and households was carried out by the survey instructors from each contact point.

**3. The selection of buildings and households in neighborhood committees /villages**

Ten residential buildings were selected by systematic sampling in each residential quarter covered by a neighborhood committee.

For each selected building, random sampling was used to the unit one of many units in a building, and then the left and right sides of the unit. households on the lower 5 floors of the selected units were surveyed.

For example: There are 123 buildings in the residential quarter covered by a municipal neighborhood committee with 10 of them being selected. The sampling interval is 123 / 10 = 12 (rounding). Then randomly select a number between 1 and 12. If for instance this is 5 then the numbers of the buildings selected will be respectively 5, 17(5+12), 29(17+12), 41(29+12) …113, etc. Next, the unit of a building is randomly selected, e.g. unit 2, then the left/ right side of the unit is chosen at random. Typically, several households are are captured at that level of sampling, for example, apartments 211-215 on the left side of unit 2 If the survey ends in failure three times in a row, the households living on the opposite side can be selected instead.

Similarly, systematic sampling was also used to select households in a village. If the survey ended in failure three times in a row, adjacent households could be selected instead.

**4. Administration of the survey**

The CHSIS questionnaire used in the survey was based on the 5th National Health Interview Survey of Households of China conducted in 2013 [1], adjusted to concentrate on primary health care. The survey was carried out using face-to-face interviews in the homes of surveyed people by undergraduates from medical universities trained by personnel from the CNHDRC. The survey participants were permanent memebers of the household. The household-owner was asked to provide information about household members who were absent at the time of the survey.

**S2 Table. CHSIS survey questions [1] evaluated in the current report.**

| **Questions from the survey** | **Response options** |
| --- | --- |
| **Household information** |  |
| Net per capita household annual income (CNY) | 0 - 4,747  4,748 - 10,887  10,888 - 17,631  17,632 - 26,937  26,938 - 50,968  >50,968 |
| **Basic information** | |
| - - - 1. Residence(called hukou in China) | 1. Rural; 2. Urban |
| 1. Gender | 1. Male; 2. Female |
| 1. Year of birth (in form of 4 digits) | yyyy |
| 1. Month of birth (in form of 2 digits) | mm |
| 1. Level of education | 1. No education  2. primary school  3. Junior high school  4. Senior high school  5. Technical school  6. Middle technical school  7. Senior technical school  8. University or higher |
| 1. Which type of health insurance scheme? | 1. Urban employee’s health schemes  2. Urban residents’ health schemes  3. New cooperative medical scheme (NCMS)  4. Rural and urban residents’ health insurance  5. Other health insurances |
| 1. Mark on the scale how your health is today from 0=very poor to 100=very healthy | Scale, 0=very poor to 100=very healthy |
| **Health behavior** | |
| 1. Do you smoke? (If the answer is yes, continue; if the answer is “no” or “have quit”, go to Q10) | 0. No  1. Yes  2. Have quit |
| 1. During the past week, how many cigarettes did you smoke every day? | 1-10 per day  11-20 per day  21-30 per day  >30 per day |
| 1. What was the frequency of alcohol consumption during the past year? | 1. <3 occasions/week  2. ≥3 occasions /week |
| 1. In the past 6 months how often do you undertake physical activities each week? | 0. Never  1. up to 1 session/week  2. 1-2 sessions/week  3. 3-5 sessions/week  4. ≥6 sessions/week |
| **Chronic disease and awareness** | |
| 1. Have you ever been diagnosed with hypertension by a doctor? | 0. No  1. Yes |
| 1. When was the last time your blood pressure was measured? | 1. Within 1 week  2. Within 1 month  3. Within 3 months  4. Within half a year  5. Before half a year |
| 1. Have any primary health care workers provided advice for your hypertension control during the past three months? | 0. No  1. Yes |
| 1. Do you take anti-hypertensive medications according to the doctor’s advice? | 1. Everyday according to doctors’ advice  2. Sometimes  3. Never |
| 1. Was your blood pressure normal when it was last measured? | 0. No  1. Yes  2. Not clear |

**S3 Table (i-iii). Description of regression model selection.**

**Step 1: Random selection of 1 participant/household**

The study cohort comprised 9,607 participants from 7,867 households, with an average of 1.2 participants per household. To simplify modelling, a single participant was randomly selected from households, in which -more than one -person was diagnosed with hypertension. This step reduced sample size from 9,607 to 7,867 participants.

**Step 2: Bivariate regression analysis of Uncontrolled Hypertension (Table i, Step 1, column 3)**

Sixteen independent variables were tested. These included variables that defined alternative regional groupings [*East/Central/West* regions, 17 *Provinces* or 34 *Districts*) as well as other demographic variables, which had been previously linked to hypertension control [2,3].

**Step 3: Multi-variable regression analysis** (Table i, columns 4-6 & Table ii, column 2)

Multi-variable regression analysis was carried out in order to adjust the association between *Management* and *Uncontrolled Hypertension* for possible confounding from known predictors of uncontrolled hypertension. Three multivariate regression models were tested, each adjusting for a different geographical characterization:

- Model A included *District* [34 areas clustered in *Province*],
- Model B included Province [17 areas clustered in *East/Center/West*] and
- Model C included *East/Center/West* regions [3 areas].

Model A had the best goodness-of-fit (Table i, column 4) and it was selected for further testing.

**Step 4: Selection of independent variables for multi-variable regression** (Table i)

Independent variables with significant contributions to the multi-variable model (Table i, model A) were retained for further testing.

**Step 5: Test of management interaction effects** (Table ii, column 3, Model F)

The secondary aim of the study was to provide context for the main findings by exploring follow-up questions arising from the results of our study. One follow-up question was whether the effect of BPHS was consistent across the demographic categories listed in Table 3. This was explored by testing the significance of *Management* interaction effects to model fit (Table ii, column 3, Model F). Interactions between *Management* and significant independent variables were tested using the Likelihood ratio (LR) test.

A significant interaction was detected between *Management* and *District* (LR chi^2^=77.9, d.f.=33, P<0.001). If this level of significance persisted to the end of the model selection process, it would suggest that the BPHS program may not be equally successful in all regions. This was not the case. After an adjustment for sampling design effects in the revised analysis, the interaction between *Management* and *District* was of borderline significance (P=0.024; Table iii, step 6, model H). This level of significance was too low to support a meaningful difference in *Management* effects in 34 *Districts*. No interaction effects were included in the final model.

**Step 6: Adjustment for sampling design effects: cluster effects at the level of *Streets/Township* clusters and *Neighborhood committee /Village*** (Table iii, column 4)

Clustering at the level of Streets/Township clusters and Neighborhood committee /Village was tested as random effects in mixed logit models (Table iii, above). Significant random effects were detected at the level of Neighborhood committee /village (ICC=0.19; LR test χ2=46.6; p<0.001). Estimated ICC was close to zero (ICC=9.0e^-35^) at the level of Streets/Township.

**S3 Table (i). Preliminary steps in testing the impact of the BPHS program (*Management*) on hypertension control.**

Step 1: Bivariate analysis was used to test the association between *Uncontrolled Hypertension* and each independent variable (IV).

Step 2: Multi-variable logistic regression analysis was used to test for possible confounding of the effect of *Management* by other known predictors of *Uncontrolled Hypertension*. The four models in Step 2 were based on alternative geographical groupings of study participants: by District / County (model A), by Province (model B), by East/Central/West regions (model C), no geographical grouping (model D).

| **Independent Variables**  **(IVs)** | **d.f. ^a^** |  | **Step 1:** |  | **Step 2:** preliminary logistic regression models,  each adjusting for a different geographical grouping | | | |
| --- | --- | --- | --- | --- | --- | --- | --- | --- |
|  |  |  | Bivariate analysis ^b^  LR test: χ^2^ (p-value) |  | Model A  With *Districts* | Model B  with *Provinces* | Model C  with *East/ Central/Wes*t regions | Model D ^c^  No geographical grouping, |
| **Key variable of interest** |  |  |  |  |  |  |  |  |
| - Management | 1 |  | **49.0 (p<0.001)** |  | **43.1(p<0.001)** | **46.0 (p<0.001)** | **52.5 (p<0.001)** | **50.4 (p<0.001)** |
|  |  |  |  |  |  |  |  |  |
| **Potential confounders** |  |  |  |  |  |  |  |  |
| Geographic variables |  |  |  |  |  |  |  |  |
| - East/Central/West China - Province - District | 2  16  33 |  | 158.8 (p<0.001)  656.9 (p<0.001)  876.9 (p<0.001) |  | ---  ---  423.0 (p<0.001) | ---  294.4 (p<0.001)  --- | 43.3 (p<0.001)  ---  --- | ---  ---  --- |
| Otherl demographic variables |  |  |  |  |  |  |  |  |
| - Insurance - Income - Compliance - Hukou - Exercise - Education - Urban/Rural residence - Wellbeing - Age - Smoking - Alcohol - Gender | 5  5  2  1  4  7  1  1  1  4  1  1 |  | 269.2 (p<0.001)  279.6 (p<0.001)  239.7 (p<0.001)  172.0 (p<0.001)  156.9 (p<0.001)  159.3 (p<0.001)  109.0 (p<0.001)  98.6 (p<0.001)  32.3 (p<0.001)  19.0 (p=0.001)  2.2 (p=0.138)  0.1 (p=0.913) |  | 9.6 (p=0.088)  25.6 (p=0.001)  87.0 (p<0.001)  7.1 (p=0.008)  13.6 (p=0.009)  27.8 (p<0.001)  0.1 (p=0.927)  40.3 (p<0.001)  8.4 (p=0.004)  7.7 (p=0.102)  2.0 (p=0.155)  0.4 (p=0.534) | 8.1 (p=0.150)  27.7 (p<0.001)  83.0 (p<0.001)  3,9 (p=0.048)  16.8 (p=0.002)  33.0 (p<0.001)  4.4 (p=0.036)  47.7 (p<0.001)  16.3 (p<0.001)  8.7 (p=0.070)  2.8 (p=0.094)  0.5 (p=0.495) | 32.1 (p<0.001)  54.7 (p<0.001)  114.9 (p<0.001)  2.3 (p=0.132)  28.9 (p<0.001)  35.3 (p<0.001)  0.8 (p=0.374)  64.9 (p<0.001)  22.7 (p<0.001)  9.6 (p=0.047)  2.8 (p=0.093)  0.4 (p=0.518) | 44.9 (p<0.001)  57.8 (p<0.001)  132.7 (p<0.001)  2.5 (p=0.117)  26.4 (p<0.001)  39.2 (p<0.001)  0.6 (p=0.453)  83.8 (p<0.001)  29.5 (p<0.001)  8.3 (p=0.083)  2.0 (p=0.161)  0.6 (p=0.426) |
|  |  |  |  |  |  |  |  |  |
| **Full model:** IVs combined |  |  |  |  |  |  |  |  |
| Combined predictors ^d^  d.f. **^a^**  Sample size | ---  ---  --- |  | ---  ---  7,867 |  | 1202 (p<0.001)  67  7,867 | 1,073.8 (p<0.001)  50  7,867 | 822.8 (p<0.001)  36  7,867 | 779.5 (p<0.001)  34  7,867 |

^a^ Degrees of freedom

^b^ Log Likelihood ratio test R test was used to evaluate the contribution of independent variables to Bivariate Logistic regression models

^c^ Models D & E were tested in response to a request from Reviewer 2.

^d^Comparison between the null model (no predictors) and a full model

**S3 Table (ii). Test of main effects and interaction effects in a multi-variable logistic regression model of *Uncontrolled Hypertension***

| **Potential Predictors of**  ***Uncontrolled Hypertension*** | **Step 3**  **Model E**  **Main effects selected ^a^**  **from Model A (Table i)** | | **Step 4**  **Model F**  **Test of *Management* Interaction effects** | |
| --- | --- | --- | --- | --- |
|  | LR test: χ^2^ (p-value) | **d.f** | LR test: χ^2^ (p-value) | **d.f** |
| **Key variable of interest** |  |  |  |  |
| - Management | **45.2 (p<0.001)** | **1** | **104.7 (p<0.001) ^b^** | **34** |
|  |  |  |  |  |
| **Potential confounders** |  |  |  |  |
| - District - Income - Compliance - Exercise - Education - Wellbeing - Age - Smoking | 460.5 (p<0.001)  28.6 (p=0.001)  89.1 (p<0.001)  14.8 (p=0.005)  30.6 (p<0.001)  37.7 (p<0.001)  9.0 (p=0.003)  10.0 (p=0.041) | 33  5  2  4  7  1  1  4 | 520.0 (p<0.001)  28.7 (p<0.001)  86.3 (p<0.001)  14.2 (p=0.007)  29.7 (p<0.001)  40.4 (p<0.001)  8.3 (p=0.004)  10.0 (p=0.040) | 66  5  2  4  7  1  1  4 |
|  |  |  |  |  |
| **Tests of *Management* Interaction terms** |  |  |  |  |
| - District - Income - Compliance - Exercise - Education - Wellbeing - Age - Smoking | ---  ---  ---  ---  ---  ---  ---  --- | ---  ---  ---  ---  ---  ---  ---  --- | **59.4 (p=0.003)**  5.6 (p=0.343)  1.3 (p=0.524)  2.1 (p=0.722)  4.9 (p=0.678)  <0.1 (p=0.946)  1.5 (p=0.224)  6.1 (p=0.192) | **33**  5  2  4  7  1  1  4 |
|  |  |  |  |  |
| **Full model** |  |  |  |  |
| - Combined predictors ^c^ - Sample size | 1,188.4 (p<0.001)  7,867 | 58 | 1,247.8 (p<0.001)  7,867 | 91 |
|  |  |  |  |  |
| **Test of variables excluded from the model** |  |  |  |  |
| - Alcohol - Gender - Insurance - Hukou - Urban / Rural residence | 2.6 (p=0.104)  0.9 (p=0.342)  3.5 (p=0.619)  1.3 (p=0.259)  <0.1 (p=0.954) | 1  1  5  1  1 | 2.1 (p=0.148)  0.7 (p=0.407)  3.3 (p=0.650)  1.2 (p=0.273)  <0.1 (p=0.991) | 1  1  5  1  1 |

^a^ Step-down method: the contribution of each IV was tested by removing it from the full model. The change in Deviance (χ^2^) was estimated using LR tests.

^b^ Impact of combined management effects (independent and interaction) in the interaction model

^c^ Comparison between the null model (no predictors) and a full model, which included significant IVs and significant interaction terms (*Management x District*)

**S3 Table (iii) Adjustment for sampling design effects: *Streets/Township* clusters and *Neighborhood committee /Village* clusters (see Table1 [B], manuscript).** Mixed effects logistic regression modelling was used to identify significant ICC**.** Random effects were used to indicate **clustering at the levels of *Streets/Township* and *Neighborhood /Village***. The contributions of random and fixed effects were estimated using Likelihood ratio (LR) tests.

| **Potential Predictors of**  ***Uncontrolled Hypertension*** | **Test of random effects in mixed models** | | | |
| --- | --- | --- | --- | --- |
|  | **Step 5: Model G**  **Main effects** | | **Step 6: Model H**  **Includes interaction terms: Management x District** | |
|  | Model contribution ^a^ | **d.f.** | Model contribution | **d.f.** |
|  |  |  |  |  |
| **Key variable of interest** |  |  |  |  |
| - Management | **44.7 (p<0.001)** | **1** | **95.6 (p<0.001)** | **34** |
|  |  |  |  |  |
| **Potential confounders** |  |  |  |  |
| - District - Income - Compliance - Exercise - Education - Wellbeing - Age - Smoking | 227.0 (p<0.001)  26.1 (p<0.001)  81.2 (p<0.001)  12.9 (p=0.012)  29.5 (p<0.001)  40.1 (p<0.001)  8.4 (p=0.004)  10.6 (p=0.031) | 33  5  2  4  7  1  1  4 | 278.0 (p<0.001)  26.3 (p=0.001)  79.5 (p<0.001)  12.7 (p=0.013)  28.6 (p<0.001)  43.0 (p<0.001)  8.0 (p=0.005)  10.7(p=0.031) | 66  5  2  4  7  1  1  4 |
|  |  |  |  |  |
| **Interaction** |  |  |  |  |
| - Management x District | ---^c^ | --- | **50.9 (p=0.024)** | **33** |
|  |  |  |  |  |
| **Random effects** |  |  |  |  |
| - Streets / township clusters (ICC ^b^ ± s.e.) - Neighborhood committee / village (ICC ^b^ ± s.e.) - LR test [χ2 (p-value)] | 9.0e^-35^ ± 7.4e^-19^  0.19 ± 0.04  46.6 (p<0.001) | 1  1  1 | 5.8e^-36^ ± 1.2e^-19^  0.05 ±0.01  38.1 (p<0.001) | ---  1  --- |
|  |  |  |  |  |
| **Full model** |  |  |  |  |
| - Combined predictors - Sample size | 1,235.0 (p<0.001)  7,867 | 59  --- | 1,285.9 (p<0.001)  7,867 | 92  --- |
|  |  |  |  |  |
| **Test of variables excluded from the final model** |  |  |  |  |
| - Alcohol - Gender - Insurance - Hukou - Urban / Rural residence | 2.9 (p=0.089)  1.0 (p=0.327)  3.7 (p=0.593)  1.4 (p=0.245)  <0.1 (p=0.899) | 1  1  5  1  1 | 2.3(p=0.132)  0.8(p=0.375)  3.5(p=0.619)  1.2(p=0.283)  <0.1(p=0.967) | 1  1  5  1  1 |

^a^  LR test [χ^2^ (p-value)].

^b^ Intra-class correlation.

^c^ --- means not applicable.

**S1 Fig. Mediation of the effect of Management on Uncontrolled Hypertension through Compliance with medication**

Investigators are often interested in assessing whether all or part of the effect of an independent variable is indirect, that is mediated through an intermediate (Fig S1). The presence of a mediated effect can be tested by decomposition of a total effect into direct and indirect components [4].

**
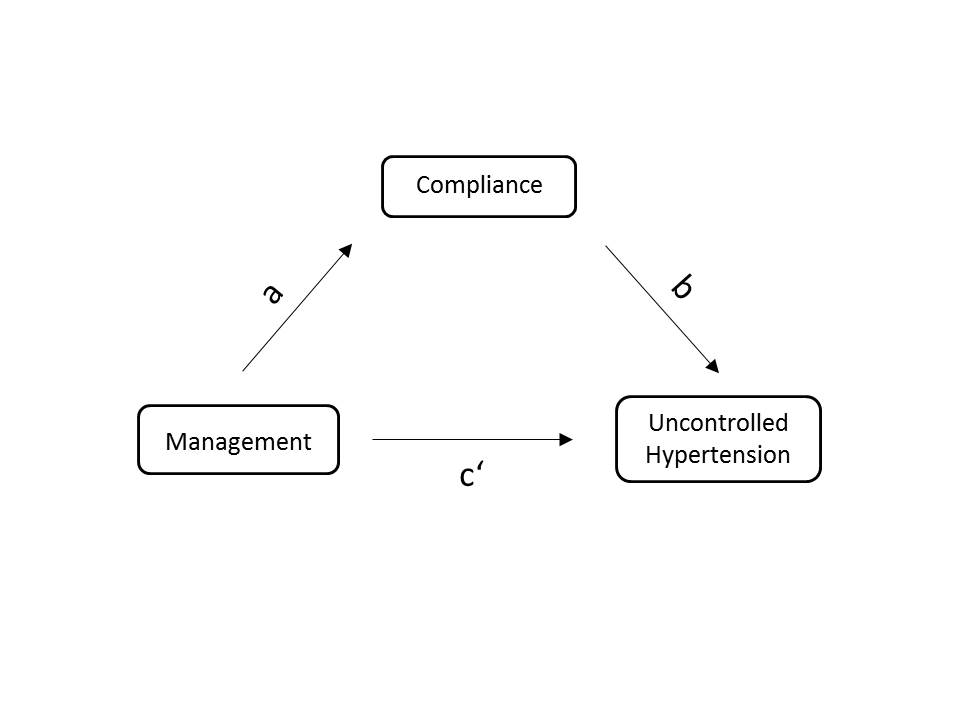
**

**S1 Fig. Path diagram illustrating the mediation of the effect of Management on Uncontrolled Hypertension through Compliance with medication. Path c’** represents the association between *Management* and *Uncontrolled Hypertension*, when *Compliance* is controlled by including it as an additional predictor of *Uncontrolled Hypertension*. **Path** **a** represents the association between *Management* and the mediator (*Compliance*). **Path b** indicates the association between the mediator (*Compliance*), and the outcome (*Uncontrolled Hypertension*).

**Mediation analysis**

We explored the possibility that the positive impact of *Management* on hypertension control among study participants might be due to improved compliance with medication among participants with appropriate PHSE follow-up. The presence of a mediated effect was tested using the product method, which employs two regression equations to break down the total effect of *Management* into direct and indirect components [5]. The direct effects of Management on Hypertension Control was estimated from parameters θ_1_ in a multi-variable mixed logit regression:

Logit{P(Y_ij_=1)} = θ_0_ + θ_1_x_ij_ + θ_2_m_ij_ + θ_3_C_ij_ + u_0j_ + ε_ij_ [1]

where x represents the treatment of interest, *Management*,

m is the potential mediator, Compliance,

C is the set of covariates described in Table 4 of the main manuscript

u0j represents Street/Township cluster-specific random intercepts and

ε is the error term.

The indirect effect of Management is indicated by parameter β_1_ in a regression of Compliance (mediator) on Management (treatment) and covariates in equation 2, which:

Logit{P(M_ij_=1)] = β_0_ + β_1_x_ij_ + β_2_C_ij_ + u_0j_ + ε_ij_ [2]

Here too, u_0j_ represents Street/Township cluster-specific random intercept (u_0j_).

The results of mediation analysis are shown in S4 Table. The direct effect is taken as θ_1_, the exposure coefficient in the outcome regression model that includes the mediator. The indirect effect is estimated as the product of β_1_ and θ_2_: the exposure coefficient in the mediator model multiplied by the mediator coefficient in the outcome model.

**S4 Table. Direct and mediated effects of Management in a model of Uncontrolled Hypertension [6].** Regression coefficient βa represents the association between the independent variable (Management) and the mediator (Compliance); βb indicates the association between the mediator (Compliance) and outcome (Uncontrolled Hypertension); θc’ represents the association between Management and Uncontrolled Hypertension. Generalised structural equation modelling (GSEM) was used to estimate the direct and indirect effects of Management. For GSEM, Compliance was recoded as a binary variable (always compliant / sometimes or never). All other variables, including covariates and random effects were the same as those listed in S3 Table (iii), above.

| Direct and indirect effects of Management | Coefficient |
| --- | --- |
|  |  |
| Regression coefficient β_a_ | 0.56 [0.39 to 0.73] |
| Regression coefficient β_b_ | -0.62 [-0.77 to -0.47] |
| Regression coefficient θ_c’_ | -0.52 [-0.67 to -0.37] |
|  |  |
| Direct effect [θ_c’_] | -0.52 [-0.67 to -0.37] |
| Indirect effect [β_a_β_b_] | -0.35 [-0.48 to -0.21] |
| Total effect [θ_c’_+ β_a_β_b_] | -0.87 [-1.06 to -0.68] |
| Proportion mediated [β_a_β_b_ /( θ_c’_+ β_a_β_b_)] | 39.9% [28.2 to 51.7] |

**S5 Table. IMS coverage per district/county.** Regional availability of IMS systems in primary healthcare care was previously reported by the China National Health Development Research Centre and the Department of Primary Care, the National Health and Family Planning Commission of China [7,8].

| **Localities** | **Established IMS ^a^**  **(%)** | |
| --- | --- | --- |
|  | **Urban** | **Rural** |
| 1. Xicheng, Beijing 2. Pinggu, Beijing 3. Yunhe, Cangzhou 4. Huanghua, Cangzhou 5. Yinzhou, Tieling 6. Xifeng, Tieling 7. Changning, Shanghai 8. Pudong, Shanghai 9. Runzhou, Zhenjiang 10. Jurong, Zhenjiang 11. Keqiao, Shaoxing 12. Shengzhou, Shaoxing 13. Meilie, Sanming 14. Shaxian, Sanming 15. Lanshan, Linyi 16. Yinan, Linyi 17. Changyi, Jilin 18. Panshi, Jilin 19. Yijiang, Wuhu 20. Fanchang, Wuhu 21. Qing Yunpu, Nanchang 22. Xinjian, Nanchang 23. Yiling, Yichang 24. Yidu, Yichang 25. Yuhu, Xiangtan 26. Shaoshan, Xiangtan 27. Hongshan, Chifeng 28. Keshiketeng, Chifeng 29. Wuhou, Chengdu 30. Xinjin, Chengdu 31. Wudang, Guiyang 32. Kaiyang, Guiyang 33. Chengbei, Xining 34. Huangzhong, Xining | 100  <100  100  <100  100  <100  100  100  100  0  100  100  100  <100  <100  100  <100  0  100  100  0  NA  <100  100  100  100  <100  100  100  100  0  NA  100  NA | NA  <100  100  100  100  <100  NA  100  NA  0  <100  <100  <100  <100  100  100  <100  0  100  100  0  100  <100  100  100  100  100  100  NA  <100  0  0  100  <100 |

^a^ Established IMS means the coverage of information management system in primary care facilities in each district or county.

**S6 Table. Comparison of Management effects between IMS groups** *Management* effects in separate *IMS* groups were obtained from three separate regression models, one for each *IMS* group: no IMS, some IMS and full IMS coverage. ^a^ the models were constructed using the same process as the one used for the regression model in Table 4. IMS availability for rural and urban regions in each district is shown in S5 Table.

| **Management groups by IMS categories** | **Number of participants** |  | **Management**  **(odds ratio [95% CI])** | **Predicted Uncontrolled Hypertension**  **( %, [95% CI])** | **Management effects** | | **Relative magnitude of Management effects** | |
| --- | --- | --- | --- | --- | --- | --- | --- | --- |
|  |  |  |  |  | **Predicted rate ratio**  **[95% CI]** | **p-value** | **Ratio of ratios**  **[95% CI]** | **p-value** |
| No IMS (0%) |  |  |  |  |  |  |  |  |
| - Unmanaged  - Managed | 206  791 |  | 1.0 a  0.96 [0.64 - 1.45] | 29.6% [22.6 - 36.6]  28.9% [24.8 - 33.1] | 1.0 a  0.97 [0.76 - 1.26] | --  0.854 | --  1.0 a | --  1.0 a |
| Partial cover (<100%) |  |  |  |  |  |  |  |  |
| - Unmanaged  - Managed | 485  1,660 |  | 1.0 a  0.58 [0.45 - 0.74] | 39.4% [34.8 - 44.1]  28.5% [26.1 - 31.0] | 1.0 a  0.72 [0.63 - 0.84] | --  <0.001 | --  0.74 [0.56 to 0.98] | --  P=0.045 |
| Full IMS cover (100%) |  |  |  |  |  |  |  |  |
| - Unmanaged  - Managed | 856  3,709 |  | 1.0 a  0.54 [0.44 - 0.67] | 28.7% [25.5 - 318]  19.8% [18.3 - 21.2] | 1.0 a  0.69 [0.61 - 0.78] | --.  <0.001 | --  0.71 [0.54 to 0.93] | --  P=0.017 |

a Differences in Management effects between IMS groups could not be tested in a single model, because the categories of an important covariate, District, were nested within IMS categories.

b Reference group.

**References for Supplementary Material**

1. Center for Health Statistics of the National Health and Family Planning Commission. An analysis report of the National Health Service 2013. Beijing: 2015 [cited 2018 Mar 15]. Available from: <http://www.nhfpc.gov.cn/ewebeditor/uploadfile/2016/10/20161026163512679.pdf>
2. Guo J, Zhu Y, Chen Y, Hu Y, Tang X, Zhang B. The dynamics of hypertension prevalence, awareness, treatment, control and associated factors in Chinese adults. J Hypertens. 2015;33(8): 1688-1696. doi: 10.1097/HJH.0000000000000594
3. Lu J, Lu Y, Wang X, Li X, Linderman GC, Wu C, et al. Prevalence, awareness, treatment, and control of hypertension in China: data from 1.7 million adults in population-based screening study (China Peace Million Persons Project). Lancet. 2017;390(10112): 2549-2558. doi: 10.1016/S0140-6736(17)32478-9
4. Valery L, VanderWeele TJ. Mediation analysis allowing for exposure-mediator interactions and causal interpretation: theoretical assumptions and implementation with SAS and SPSS macros. Psychol. Methods. 2013;18(2): 137-150.
5. Mascha EJ, Dalton JE, Kurz A, Saager L. Understanding the mechanism: mediation analysis in randomised and nonrandomised studies. Anesth Analg. 2013;117(4): 980-994.
6. Rijnhart JJM, Twisk JWR, Eekhout I, Heymans MW. Comparison of logistic-regression based methods for simple mediation analysis with a dichotomous outcome variable. BMC Med Res Methodol. 2019;19: 19. https://doi.org/10.1186/s12874-018-0654-z
7. China National Health Development Research Center and the Department of Primary Care, National Health and Family Planning Commission of China. Monitoring and survey report of the 34 key contact pilot areas for primary care comprehensive reform of China. 29 Oct 2015 [cited 2017 Jul 25]. Preprint. In Chinese.
8. Zhang YC, Qin HM, Yang XQ, Zhang LF, Lin CM. Risk prediction of cardiovascular and cerebrovascular diseases on community patients with chronic disease in Beijing. Chinese Gen Pract. 2017;20(5): 590-594. doi:10.3969/j.issn.1007-9572.2017.05.017
